# Advances in Serological Diagnosis of *Taenia solium* Neurocysticercosis in Korea

**DOI:** 10.5808/GI.2019.17.1.e7

**Published:** 2019-03-31

**Authors:** Chun-Seob Ahn, Jeong-Geun Kim, Sun Huh, Insug Kang, Yoon Kong

**Affiliations:** 1 Department of Molecular Parasitology, Samsung Medical Center, Sungkyunkwan University School of Medicine, Suwon 16419, Korea; 2 Department of Parasitology and Institute of Medical Education, Hallym University College of Medicine, Chuncheon 24252, Korea; 3 Department of Biochemistry and Molecular Biology, Kyung Hee University College of Medicine, Seoul 02447, Korea

**Keywords:** immunodiagnosis, neurocysticercosis, proteome, Republic of Korea, *Taenia solium*

## Abstract

Cysticercosis, a parasitic disease caused by *Taenia solium* metacestode (TsM), has a major global public health impact in terms of disability-adjusted life years. The parasite preferentially infects subcutaneous tissue, but may invade the central nervous system, resulting in neurocysticercosis (NC). NC is an important neglected tropical disease and an emerging disease in industrialized countries due to immigration from endemic areas. The prevalence of taeniasis in Korea declined from 0.3%–12.7% during the 1970s to below 0.02% since the 2000s. A survey conducted from 1993 to 2006 revealed that the percentage of tested samples with high levels of specific anti-TsM antibody declined from 8.3% to 2.2%, suggesting the continuing occurrence of NC in Korea. Modern imaging modalities have substantially improved the diagnostic accuracy of NC, and recent advances in the molecular biochemical characterization of the TsM cyst fluid proteome also significantly strengthened NC serodiagnosis. Two glycoproteins of 150 and 120 kDa that induce strong antibody responses against sera from patients with active-stage NC have been elucidated. The 150 kDa protein showed hydrophobic-ligand binding activities and might be critically involved in the acquisition of host-derived lipid molecules. Fasciclin and endophilin B1, both of which play roles in the homeostatic functions of TsM, showed fairly high antibody responses against calcified NC cases. NC is now controllable and manageable. Further studies should focus on controlling late-onset intractable seizures and serological diagnosis of NC patients infected with few worms. This article briefly overviews diagnostic approaches and discusses current issues relating to NC serodiagnosis.

## Introduction

Cysticercosis is a parasitic disease caused by infection with the larval stage of the cyclophyllidean tapeworm *Taenia solium*. The *T. solium* metacestode (TsM) preferentially lodges in subcutaneous tissues and muscles, but often invades the central nervous system (CNS), resulting in neurocysticercosis (NC). NC, which is a leading cause of adult-onset seizure disorder, is a major global public health concern in several endemic areas [1]. Annually, NC causes approximately 28,000 deaths and more than three million people are at risk [2]. In endemic areas of Latin America and Africa, NC accounts for 10%–12% of all hospital admissions to neurological departments [3]. NC is becoming an emerging or a re-emerging disease in industrialized countries, due to the high frequency of immigration from endemic areas. Moreover, NC is now regarded as one of the most important food-borne zoonotic helminthiases [4].

Historically, taeniasis and cysticercosis have posed formidable public health problems in Korea. In the 1970s, the prevalence of taeniasis was 0.3%–12.7% according to stool examinations and 4.5%–38.0% by questionnaire surveys [5]. However, the prevalence is currently below 0.02%, and most affected patients are elderly and originate from remote islands [6]. From 1987 to 1990, a survey investigated the seroprevalence of NC in mixed epilepsy patients in Korea. A total of 2667 serum samples, randomly selected from 27 different localities, were tested for their specific antibody levels by enzyme-linked immunosorbent assay (ELISA). The positivity rate was 4.0% in the patient group (standardized positivity rate, 3.1%) and 2.1% in persons without mixed epilepsy (standardized rate: 1.8%). Geographically, the rate was the highest in patients living in Jeju Province (8.4%). Patients aged 50–59 years showed the highest positivity rates [7]. Another study of surgical specimens revealed that 149 of 80,947 cases (0.18%) were of parasitic origin. Of these, 112 cases were cysticercosis involving several organs, most commonly subcutaneous tissues and muscles [8]. From 1993 to 2006, a total of 74,448 serum samples obtained from patients with neurological manifestations and liver diseases were tested by ELISA. The percentage of samples showing high antibody titers against TsM cyst fluid (CF) antigen decreased from 8.3% to 2.2% [9], which indicated that cysticercosis, including NC, has continued to occur in Korea, although its incidence has substantially decreased.

The symptoms of NC vary depending on the number, location, and size of the infected worm(s); the duration of infection; the evolutionary stage of lesions; and the presence or absence of acephalic budding cysticercus [10,11]. Major symptoms include headache, seizure, paresis, and focal neurologic deficits. More importantly, NC is a leading cause of adult-onset seizure in areas where the disease is endemic [1,12].

The accurate diagnosis of NC is important for effectively controlling the disease. The key to the diagnosis of NC is interpreting the patient’s manifestations together with data provided by radiological findings and immunological tests, since the parasite’s eggs are not present in the patients’ stool. Neuroimaging diagnosis depends largely on computed tomography (CT) and/or magnetic resonance imaging (MRI) because a definitive histological diagnosis is neither possible nor feasible in many cases [13,14]. However, these methods have limitations due to the highly pleomorphic presentation of NC, overlapping with that of other intracranial space-occupying lesions [14,15]. Tests to detect specific antibodies in the patient’s serum/cerebrospinal fluid (CSF) have been applied to provide additional evidence. Immunodiagnosis might support other diagnostic procedures for NC in neurological patients [16,17].

Many cases of active-stage NC can be treated with specific chemotherapeutics. However, chronic NC and acephalic cysticercosis in the ventricles are not susceptible to anthelminthics, but should instead receive surgical or symptomatic treatment, such as worm removal surgery, anticonvulsants, analgesics, or shunt operations to relieve the increased intracranial pressure or to control intractable seizures [18]. Specific treatment can shorten and diminish the symptoms caused by inflammation associated with active NC, and such treatment appears to reduce the risk of headache and late-recurring refractory seizure [1,18].

In Korea, the mid-1980s brought dramatic advances in the management of NC. Emerging neuroimaging techniques greatly improved the capability to diagnose organic brain diseases [13,19]. The concomitant application of serological diagnoses [20,21] provided further evidence for the presence of the worm in the brain. Safe and effective chemotherapeutics for the medical treatment of NC were successfully launched [22,23]. Several molecular and cell biological studies have elucidated bioactive molecules of TsM that are crucially involved in the regulation of homeostatic functions [24,25] and the symbiotic protein interactome network of the worm [26]. These efforts have made the disease controllable and manageable. However, unsolved problems remain for the more effective management of the disease, such as control of intractable seizures, especially after medical treatment; the diagnosis of acephalic cysticercosis in the ventricles; and the serological diagnosis of early infections and patients who are infected with relatively few worms in the CNS and other areas. In this article, we briefly overview the advances and current status of the immunodiagnosis of NC in Korea.

## Assessment of Serodiagnosis of Cysticercosis

As a first step toward NC serodiagnosis, an extensive study to determine the applicability of ELISA employing TsM CF as an antigen was carried out using paired samples of serum and CSF from patients with NC. The overall sensitivity and specificity were 90.1% and 88.5%, respectively. CSF showed greater sensitivity. This result demonstrated that a serological test using paired samples of serum and CSF was highly beneficial for differentiating NC from other causes of organic brain diseases [20].

To establish the appropriate diagnostic criteria, the specific antibody levels were measured in 1:100 diluted serum (in phosphate buffered saline containing 0.05% Tween 20) and undiluted CSF samples from 355 patients with several neurological diseases, including NC. The positive criterion was set as the low limit of the positive reaction, which was also the mid-point of the absorbance range of the lowest frequency in serum samples used in the blind test [20]. Considering that the amount of specific IgG might be 1/100 of the amount in serum [27], the same positive criterion could be applied for CSF samples. Detection of specific antibody levels by ELISA either in serum or in CSF, thereafter, became the main mode of NC serodiagnosis. IgG subclass responses demonstrated that sera from patients with NC reacted mainly with the IgG4 subclass [28].

## Identification of the Immunopotent Component of TsM for NC Serodiagnosis

One of the critical problems encountered in the serological diagnosis of NC is the differential diagnosis from other parasitic infections. Cross-reactions that arise from other larval cestodiases, such as alveolar and cystic echinococcoses (AE and CE, respectively), and sparganosis should be ruled out, since these larval cestodiases frequently provoke serological cross-reactions [29].

**Fig. 1.**
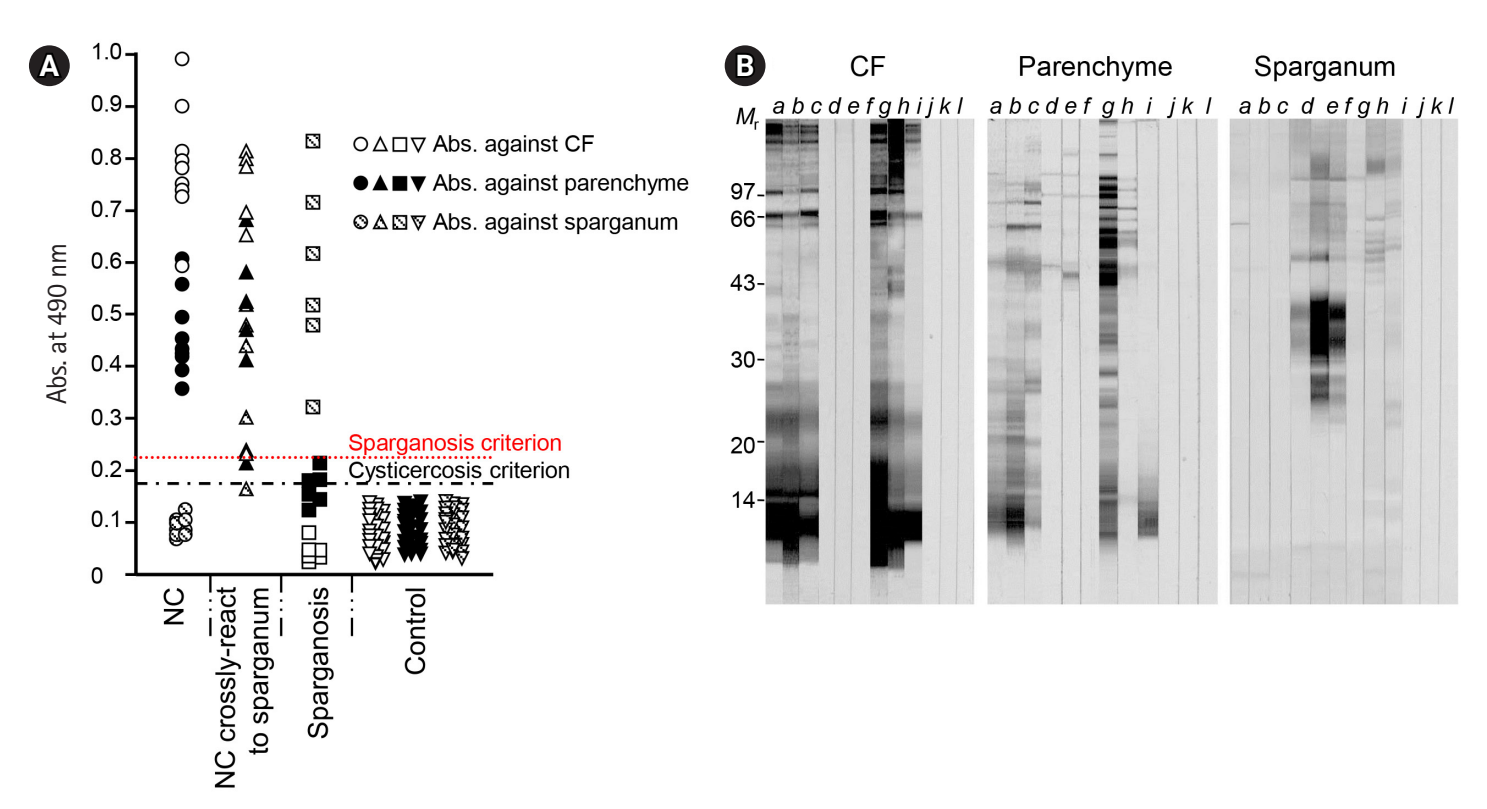
(A) Scattergram of specific antibody levels in sera from patients with NC, NC crossreacted to the sparganum extracts and sera from patients with sparganosis. Serum samples from NC cases showed the highest levels of reactivity against TsM CF. Some sparganosis sera revealed cross-reactions against TsM parenchymal extracts, but showed no crossreactions against TsM CF. (B) Immunoblot analysis of three different antigens (TsM CF, TsM parenchymal extracts, and sparganum extracts). Lanes a–c, sera from NC patients; lanes d–f, sera from sparganosis patients; lanes g–i, sera from NC cases cross-reacted with the sparganum extracts, lanes j–l, negative controls. NC, neurocysticercosis; TsM, *Taenia solium* metacestode; CF, cyst fluid; *M*_r_, molecular weights in kDa.

For the serological differentiation of NC from sparganosis, the diagnostic properties of TsM CF proteins were compared with those of parenchymal extracts. CF showed a higher antibody detection capability than parenchymal extracts. Sera from NC patients cross-reacted with the sparganum extracts, and the TsM antigen showed cross-reactions to the sera of patients with sparganosis. Approximately 50% of the sera from sparganosis patients exhibited cross-reactions against TsM parenchymal extracts (Fig. 1A and 1B). This finding suggested that some NC patients might produce IgG antibodies against TsM antigenic proteins, which are common in sparganosis and non-specifically bind to sparganum extracts. The antigenic components that caused the cross-reaction were not a single protein, but multiple proteins (Fig. 1B).

When sera from NC patients were examined by immunoblot, TsM CF exhibited a variety of reaction patterns. Several protein bands revealed positive antibody responses, of which the bands at 7, 10, 15, 20–40, 43, 64, 95, 106, and 160 kDa were strongly reactive (Fig. 1B, panel CF). The 10 kDa component showed the strongest reaction, with 84.6% of the samples (209 of 247) being positive [28]. Although the reaction pattern of the parenchymal extracts was similar to that of the CF, more numerous bands ranging from 43 to 100 kDa were reactive with sera from NC patients. In general, high-molecular-weight proteins above 47 kDa were shown to be cross-reactive both with TsM parenchymal extracts and with sparganum extracts, while the low-molecular-weight proteins (LMWPs) (ranging from 7 to 24 kDa) in CF showed specific antibody reactions. Our research group observed similar results for serological cross-reactivity with AE and CE. High-molecular-weight proteins typically revealed cross-reactions [28,29]. Those antigenic bands did not seem to be the same molecules as the major antigenic proteins of TsM. This result further suggested that TsM CF is suitable for the differential diagnosis of cysticercosis from other cestodiases, as well as better than parenchymal extracts for the serodiagnosis of cysticercosis [20,28].

## Two Macromolecular Proteins of TsM CF Display Specific Antibody Reactions against Sera from NC Patients

A study was conducted to purify antigenic components from TsM CF by monoclonal antibody-ligand immunoaffinity chromatography. The purified protein migrated as a single homogenous band by native gel electrophoresis and was further separated into three subunits of 7, 10, and 15 kDa by sodium dodecyl sulfate–polyacrylamide gel electrophoresis (SDS-PAGE) analysis. The diagnostic significance of the purified protein was evaluated by ELISA. The specific IgG antibody levels in serum and CSF samples from NC patients were not higher than the levels in crude CF [30]. This result indicated that the purified protein had higher specificity, but lower sensitivity, as a diagnostic antigen, which may have been partially due to its limited detection of mono-specific or oligo-specific antibodies circulating in the patients’ sera among the diverse polyclonal antibodies produced in the patients.

**Fig. 2.**
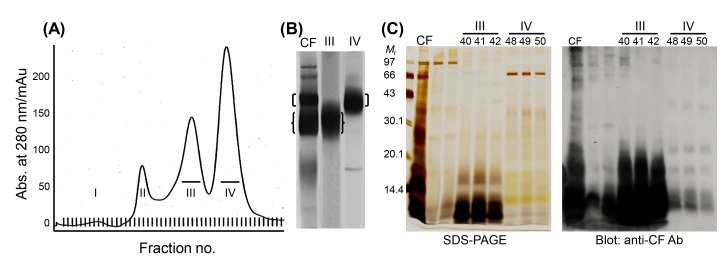
Partial purification of 120 and 150 kDa macromolecular proteins from TsM CF. (A) A 1.6 × 60 cm long FPLC packed with Superdex 200 prep grade was equilibrated with 20 mM Tris-HCl (pH 8.0) containing 150 mM NaCl. A total of 3 mL (10 mg) of TsM CF was applied to the column with a flow rate of 0.5 mL/min. Eighty-five fractions (1.5 mL each) were analyzed for their absorbance at 280 nm, as monitored by UNICORN (v3.0). (B) Analysis of the eluted group III and IV proteins by native PAGE. (C) Western blot analysis of the fraction III and IV proteins probed with anti-CF antibody. TsM, *Taenia solium* metacestode; CF, cyst fluid; FPLC, fast-performance liquid chromatography; PAGE, polyacrylamide gel electrophoresis; Mr, molecular weights in kDa; SDS-PAGE, sodium dodecyl sulfate–polyacrylamide gel electrophoresis.

Another series of experiments to characterize LMWPs was carried out through a proteomic analysis combined with gel permeation chromatography. When eluted with Superdex 200 fast-performance liquid chromatography, TsM CF revealed four major peaks (Fig. 2A), in which fractions III and IV constituted the major groups. Fractions III and IV harbored a 150 kDa and a 120 kDa macromolecular protein, respectively (Fig. 2B). The proteins were further divided into several LMWPs that showed high antibody responses against anti-CF antibody (Fig. 2C). These molecules are members of the major groups of LMWPs that have been extensively studied owing to their potent antigenic properties [28,31-33].

**Fig. 3.**
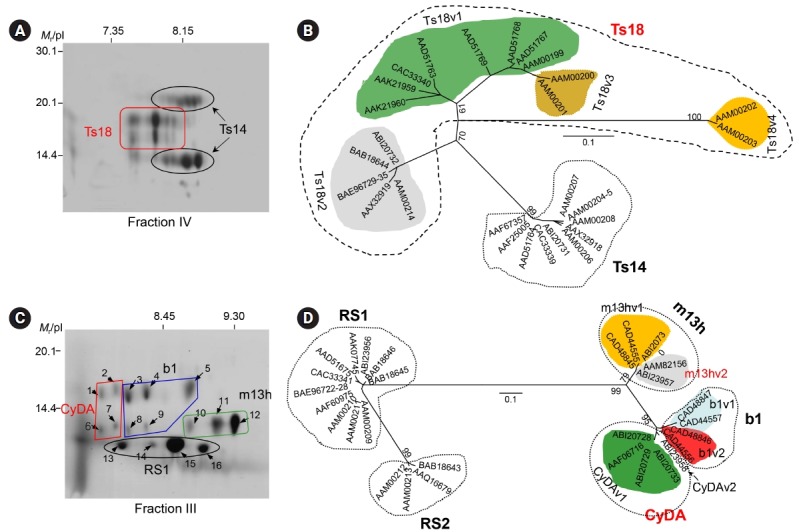
Biophysical properties and phylogenetic relationship of the 120 and 150 kDa proteins of the TsM CF. (A) The 120 kDa protein (fraction IV) was separated in a pH range of 6–11, after which it was further resolved by 15% SDS-PAGE. The subunit proteins of 14, 16, 18, 22, and 28 kDa, which were identified by proteome analysis, are seen. (B) Phylogenetic analysis demonstrated that the subunits of the 120 kDa macromolecule were clustered into two groups of 14 and 18 kDa lineages. (C) 2DE analysis of the 150 kDa protein (fraction III). The protein was isoelectrically focused using IPGphor (pH 6–11), after which it was further separated by 15% SDS-PAGE. Each protein was subjected to MALDI-TOF analysis. (D) Phylogenetic analyses of the subunit proteins. The representative amino acid sequences were aligned, and optimized with ClustalW and GeneDoc. The phylogram was constructed with the neighborjoining algorithm (PHYLIP). The statistical significance of each branching node was evaluated with 1,000 random samples. TsM, *Taenia solium* metacestode; CF, cyst fluid; SDS-PAGE, sodium dodecyl sulfate–polyacrylamide gel electrophoresis; 2DE, two-dimensional electrophoresis; MALDI-TOF, matrix-assisted laser desorption ionization time-of-flight; *M*_r_, molecular weights in kDa.

We characterized the 120 kDa macromolecule (fraction IV) (Fig. 2B). The protein consisted of two major components of 42–46 and 22–28 kDa when analyzed using non-reducing SDS-PAGE gels and shared three subunits of 14, 16, and 18 kDa in the results of reducing SDS-PAGE analysis. The 42–46 kDa component contained three additional subunits of 22, 28, and 38 kDa [34]. These molecules were linked by intra-/inter-subunit disulfide bonds. Two-dimensional electrophoresis (2DE) showed that each of these subunit proteins might be the result of post-translational modifications, such as glycosylation and phosphorylation (Fig. 3A). Cloning and phylogenetic analysis of these molecules established that these six subunits potentially originated from either the 14 or 18 kDa precursor (Fig. 3B), which might have consisted of heterozygous molecules. Our research group assessed the antibody reactivity of the native protein and the recombinant 14 and 18 kDa proteins and observed that both the native and recombinant proteins had a high reliability for differentiation of active- and mixed-stage NC from chronic NC [34]. Interestingly, the derivatives of the 18 kDa subunits displayed different isoelectric point values and were shown to be abundantly detected in the CF collected from the Americas, but the opposite was evident for the 14 kDa subunits [17]. Immune recognition patterns against the 120 kDa protein also varied according to the geographic origins. The reactions were more distinct and prominent in CFs of Central/Latin American origin than those of Asian origin [17].

We also investigated the biochemical, biophysical, and immunological properties of the 150 kDa protein (fraction III) (Fig. 2B). The protein was found to be a hetero-oligomeric complex consisting of multiple subunits of 7, 10, and 15 kDa within the pH range of 8.0–9.7. The subunits may have originated from four unique genes of 7 and 10 kDa gene families with 2–3 polymorphic alleles/paralogs (Fig. 3C). The 10 kDa group had diverged from CyDA1 and 2, b1 variant (b1v) 1 and 2, and m13h variant (m13v) 1 and 2. The 7 kDa proteins were separately clustered with RS sublineages (RS1 and RS2) (gene names were adapted from GenBank DB) (Fig. 3D). However, we could not identify the RS2 lineage through the 2DE analysis. The 15 kDa protein represented a glycosylated form of the 10 kDa protein [24]. These subunit proteins showed highly specific antibody reactivity against sera from NC patients, but were not reactive with the sera from patients with other parasitic infections, including AE, CE, and sparganosis [17]. Epitope mapping of the 10 kDa subunit, which showed the most reliable diagnostic performance, revealed that amino acid residues 30–34—Asn-Met-Thr-Val-Met (NMTVM)—comprised the core sequence of the dominant epitope [35]. In contrast to the 120 kDa protein, the expression characteristics of the 150 kDa molecule did not significantly differ between Asian and Latin American samples of TsM. This result further suggested that 150 kDa molecule was better for detecting specific antibodies circulating in the patient sera, without genomic drift compared to the 120 kDa protein [17]. Additional studies are warranted to determine the involvement of each subunit in antibody recognition, the molecular mechanism that controls the transcriptional/translational expression of these subunits, and the manner in which these subunits are linked to form tertiary and quaternary structures.

Our research group also investigated the biological function of the 150 kDa protein. An ex vivo experiment employing viable TsM demonstrated that the excreted protein bound to lipids and participated in the uptake of lipids from the surrounding host tissues. The process was substantially inhibited by specific anti‐150 kDa antibodies. The protein was localized in the parasite syncytium and in the lipid droplets within the host granuloma wall, where significant lipase activity was expressed [24]. The subunits comprising the 150 kDa protein indeed revealed high binding affinity to lipid analogs with non-overlapping patterns. The protein showed evidence of high-level sequence identity with other cestode hydrophobic-ligand binding proteins (HLBPs) and formed a novel clade associated with excretory-secretory HLBPs. A study with mutagenic RS1 proteins demonstrated that structural/electrostatic integrity around the second α-helix, rather than the conventional Trp residue, was the major factor governing the hydrophobic interaction [36]. HLBP-mediated uptake of the host lipids may be critical for the survival of the parasite and maintenance of parasitic homeostasis; therefore, therapeutics or vaccines might be exploited by interruption of the biological functions of this protein [24].

## Antigen Cocktail and Chimeric Antigen for the Specific Diagnosis of Early Active-Stage NC

**Fig. 4.**
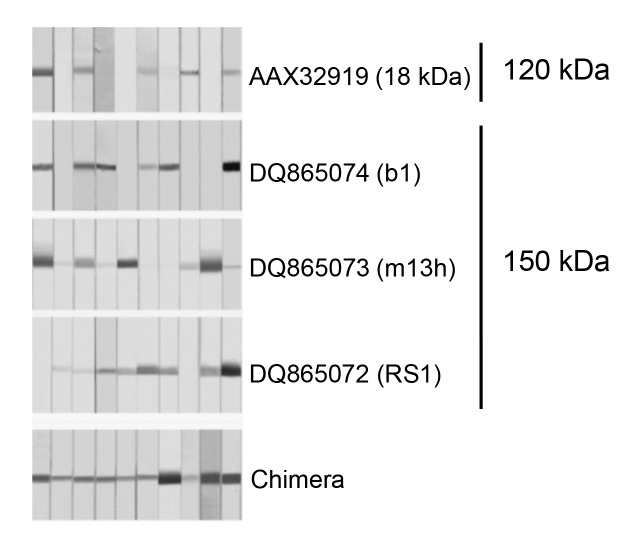
Immune response of the representative recombinant proteins derived from the 120 and 150 kDa subunits and chimera against sera from active-stage NC patients. Each protein was separated by 12% SDS-PAGE and transblotted to a PVDF membrane. The blots were incubated with 1:200 diluted individual NC sera overnight. The blots were further incubated with horseradish peroxidase–conjugated anti-human IgG and developed with 4-chloro-1- naphthol as a chromogen. NC, neurocysticercosis; SDS-PAGE, sodium dodecyl sulfate–polyacrylamide gel electrophoresis; PVDF, polyvinylidene difluoride.

We cloned four representative proteins (one from the 120 kDa protein and three from the 150 kDa protein) that showed different epitope specificities, and an antigenic cocktail and a chimeric protein were constructed by fusing catenated genes. These regimens had a great deal of potential to facilitate the development of novel standard diagnostic assays, as this protein had the advantage of compensating for epitope specificity (Fig. 4). The overall sensitivity and specificity of the chimera was determined to be 97.5% (156 of 160 samples) and 97.8% (265 of 271 cases), respectively [17]. The antigenic cocktail also showed similar results. More studies employing large numbers of serum and CSF samples may be essential to validate the practical value of these regimens. Identifying and compiling other novel antigenic molecules that bear different epitope specificity will further contribute to the design of final platform regimen(s).

## Endophilin B1 and Fasciclin Proteins of TsM Show High Antibody Reactivity against Sera from Chronic Inactive NC Cases

Endophilin B1 plays critical roles in the maintenance of membrane coverture and endocytosis in living organisms [37]. We isolated three proteins homologous to endophilin B1 from three human-infecting Taenia species (sequence identity, 92.9%–96.6%). Taeniidae endophilin B1 showed a unique immunological profile and was abundantly expressed in the tegumental syncytium of the larval and adult worms. Taeniidae endophilin B1 might be involved in the control of membrane dynamics, thereby contributing to shaping and maintaining the tegumental curvature. The protein was secreted into the host tissue and induced strong host immune responses, especially against sera from patients with chronic NC, CE, and AE. Bacterially expressed recombinant *T. solium* endophilin B1 (rTsMEndoB1) also revealed similar results. It demonstrated a sensitivity of 79.7% (345 of 433 cases) for serodiagnosis of larval Taeniidae infections. The characteristic patterns of TsMEndoB1 expression may be related to the antibody responses observed in chronic cases. Endophilin B1, as a major constituent of the tegument, may be continuously expressed during the involution process of the parasites, although its expression levels decline over time. It may continually stimulate the host immune systems to produce specific antibodies until the parasites are completely regressed or calcified. Use of rTsMEndoB1 might not be suitable for the initial diagnosis of NC patients, but could be beneficial for follow-up surveillance of NC [38].

We also isolated two paralogous fasciclin-like molecules (TsMFas1 and 2) that demonstrated fairly high antibody responses against sera from chronic NC patients. These molecules had molecular weights of 83 kDa (secretory form, 65 kDa) and harbored fasciclin and fasciclin‐superfamily domains. The proteins were shown to be expressed as multiple isoforms by post-translational phosphorylation. The proteins were constitutively expressed in metacestode and adult stages, with preferential locality in the scolex. Bacterially expressed rTsMFas1 exhibited 78.8% sensitivity (63 of 80 cases) and 93% specificity (278 of 299 samples) in diagnosing chronic NC. Although TsMFas1 demonstrated some cross-reactions with sera from CE patients (10 of 56, 17.8%) and sparganosis (4 of 50, 8%), TsMFas1 may be useful for the differential diagnosis of chronic NC in clinical settings, especially where both NC and other infectious cerebral granulomatoses are prevalent. The expression pattern of these molecules in the scolex was also found to be related to the antibody reactivity that culminated in chronic cases, as evidenced by endophilin B1 [39]. In addition, TsMFas molecules bound to calcareous corpuscles may symbiotically mediate protein-protein interactions governing carbohydrate-metabolizing enzymes and cytoskeleton/cell motility to maintain efficacious homeostatic functions and ensure the prolonged survival of the TsM in the host [26].

## Detection of the 150 kDa Protein in Serum and CSF Samples from NC Patients

The amount of the 150 kDa protein in CF was measured by antibody-sandwich ELISA in serum and CSF samples from NC patients. The capture antibodies were a rabbit anti-CF antibody and a monoclonal antibody against the 150 kDa protein. The detection limit of the test was 8 ng/mL of the protein. The levels of the protein in 351 sera from 255 patients (55 surgery-proven cases and 200 cases diagnosed by positive antibody reactions and pathognomonic CT/MRI findings) were below the sensitivity of the assay. Of the 276 CSF samples tested, 31 (11.2%) revealed a detectable range, which indicated that the detection of antigenemia might not be sensitive enough to diagnose NC. The 150 kDa protein appeared in the CSF in situations such as 2 days after praziquantel treatment, or a patient infected with a racemose cysticercus with a degenerated cyst wall. The appearance of free 150 kDa protein might be associated with cyst wall rupture. Either by drug treatment or by natural degeneration of old TsM, CF proteins might be released into the CSF [40].

## Serological Follow-up Monitoring of NC

**Fig. 5.**
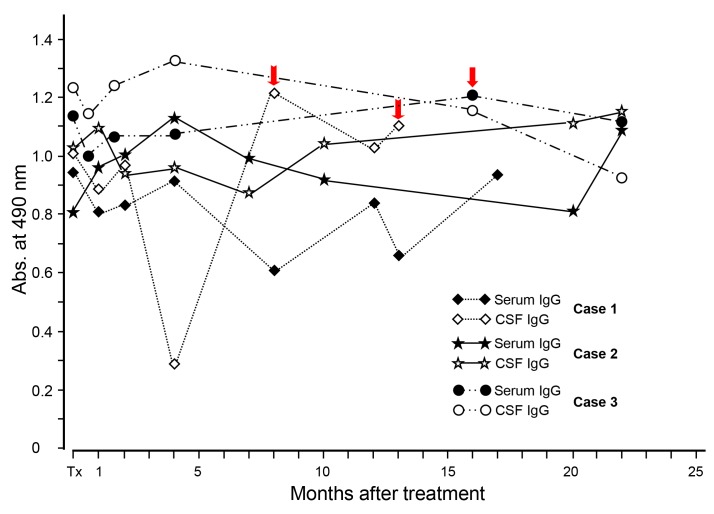
Changing patterns of specific IgG antibody levels in serum and CSF samples from the NC cases after medical treatment shown by individual patients. Acute encephalitic attacks in each patient are indicated by red arrows. Significant changes of specific IgG antibody levels in either serum or CSF were not observed during the 22-month observation period. CSF, cerebrospinal fluid; NC, neurocysticercosis.

Our research group assessed changing patterns of specific antibody levels after medical treatment. A total of 69 patients with NC participated in the study. The intervals and number of examinations during the follow-up period varied according to the patient (up to 22 months). Serially-collected serum and CSF samples were examined simultaneously for their specific IgG antibody levels by ELISA using a CF antigen. Individual patients showed a typical pattern of specific IgG antibody levels after treatment (Fig. 5). Within four-month post-treatment, the antibody levels were temporarily elevated in both the serum and CSF in most patients. However, in some cases, antibody levels steadily declined after treatment. Some samples that showed a negative antibody reaction before treatment turned out to be positive after treatment. This result suggested that follow-up examinations could be beneficial as a complementary tool (provocation test) in serodiagnosis. One to two months were considered to be a sufficient interval for initial follow-up surveillance for that purpose [41]. However, at the 22-month follow-up, most NC patients with a long history did not show any significant changes in specific antibody levels in either the serum or CSF. These results demonstrated that yearly serologic follow-up should be continued for at least 5 years after treatment to differentiate cured patients from chronic patients with slowly calcifying lesions.

## Concluding Remarks

NC, a parasitic disease caused by infection of the CNS with the TsM, poses formidable global public health problems and critically affects disability-adjusted life years. NC has become an emerging or a re-emerging disease in industrialized countries due to an increasing frequency of immigration from endemic areas. The disease is now regarded as one of the most important emerging diseases, together with other viral- and drug-resistant bacterial infections [42].

NC caused public health concerns in Korea over the past several decades. However, dramatic improvements in control and management took place during the mid-1980s. The introduction of CT and MRI greatly increased the diagnostic accuracy, and the development of serodiagnostics provided supportive or confirmatory evidence for NC. Several investigations have shown that the detection of LMWPs of the 150 kDa and 120 kDa subunits in either serum or CSF is a good target for the diagnosis of active-stage NC. Recent progress in NC serodiagnosis has resulted in two different types of antigenic platforms. One is a chimeric protein fused with defined molecules with different epitope specificities [17], and the other is a multi-antigen print immunoassay that uses different antigens as a cocktail [17,43]. Both of these regimens exhibited reliable diagnostic feasibility, especially for the diagnosis of active-stage NC. Diagnosing active NC is important because it allows treatment with specific chemotherapeutics, while chronic-stage or acephalic budding cysticercosis in the ventricles is not subject to anthelminthics, but requires surgical or symptomatic treatment to control intractable seizures.

All these efforts have made NC controllable and manageable. However, further study is required to find solutions for the control of late-onset intractable seizures, especially after medical treatment, and for the serological diagnosis of NC patients infected with few worms. Such further advances would enhance the detection, management, and prevention of NC. The identification and combing of other novel antigenic molecules that bear different epitope specificity will further contribute to the design of final platform regimen(s).
